# Rate-Supported Hospitals

**Published:** 1898-05-21

**Authors:** 


### The Hospital

A Journal of

The Medical Sciences and Hospital Administration.

Vol. XXIV.—No. 608.] Edited by Sir Henry Burdett, K.C.B., and Solomon C. Smith, M.D., M.R.C.P. [May 21, 1898.

### Rate-Supported Hospitals.

It is now some years since a large tank on the roof of St. George's Hospital suddenly gave way, and permitted many tons of water to escape with a rush, which carried all before it, which literally cut out a square of considerable size from three successive floors, carried down beds and patients in one common ruin, washed a few students out of their sitting-room into the area, and led to the loss of more than one life. A well-known and witty Irish nobleman who, a few days after the event, was walking westward in Piccadilly, was met by a gentleman of great wealth but of strict economy, who stopped him to discourse about the accident, and to express his inability to understand how it had occurred. The nobleman asserted that the causes were evident upon the surface, and that, if his friend would turn and walk back with him he would show them. The friend was incredulous, said that he had himself been on the roof of the hospital that morning, and that he was still entirely puzzled. He turned back, and, when they came close to the building, the nobleman said, "There now, you have only to look up and you will see how it happened." "I see nothing," was the reply. "Sure, look again," answered the nobleman. "Don't you see 'Supported by Voluntary Contributions,' and the support wasn't sufficient, that's all!" Even where tanks do not thus take upon themselves to act as monitors to benevolence, there is no doubt that insufficiency of support is the great blot upon the voluntary system.

Still, it by no means follows that the reverse of wrong is right; and, while it is incontestable that a rate in aid or in support of hospitals would be an effectual means of extracting money from the pockets of persons who ought to be subscribers but are not, it is only too certain that the control of hospital administration by the persons who usually control the expenditure of rates would not necessarily be conducive to efficiency. Thirty years ago, the condition of workhouse infirmaries became a public scandal; and, although many improvements have since been introduced into them, they are still, as a rule, very inferior to voluntary hospitals in all their arrangements for the care and treatment of the sick. A rate-supported hospital would, of course, be upon a large scale; and no hospital on a large scale can be efficient unless it is connected with a medical school, and is able to command, in various departments and in various ways, the assistance of medical students. The hospitals controlled by the Metropolitan Asylums Board are, in many respects, models for imitation; but their usefulness is almost entirely limited to the benefits which they confer upon the patients actually received within their walls.

The usefulness of St. George's, or of St. Bartholomew's, is not thus limited, but extends in ever widening circles, on account of the knowledge which they afford to students, and which the students, in due time, come to apply in all parts of the world. Every patient who is rescued from death or from pain by medical assistance, owes a debt of gratitude, not only to the physician or surgeon immediately concerned in curing him, but also to the hospital at which that physician or surgeon acquired his skill; so that what many people would regard as only a secondary object of hospitals, the distribution of medical science over the world, should rather be regarded as the primary one, because it beneficially affects a far larger number of people.

Now, a medical school cannot be created by the fiat of ratepayers, but only by the gradual accumulation of students around teachers of proved capacity; and hence, assuming the voluntary and the rate-supported hospital to be maintained at equal expense, the latter would be extravagant as compared with the former, because it could only accomplish one portion of the former's work. It is perfectly right that hospitals which deal with infectious diseases should be supported by rates, because their primary object is to secure the safety of the public; but when we come to diseases which are not of this character, and from which the only sufferers are the persons afflicted by them, then a greater elasticity of management than rates will usually afford seems to be imperatively required. Even as it is, the wishes of the medical officers, wishes guided by knowledge and dictated by a desire to do the best that is possible for the patients and for the teaching, are not always met in a proper spirit by non-medical governors; and the interests of science would be likely to fare badly in any institution in which these governors, who, after all, usually owe their positions to a sincere, if not necessarily intelligent, desire to do their best, were superseded by a committee of guardians, chosen at random after an election which had turned upon some such question as the desirability of a fresh coat of paint upon the railings around the parish cemetery.

